# Evaluating public involvement in research design and grant development: Using a qualitative document analysis method to analyse an award scheme for researchers

**DOI:** 10.1186/s40900-016-0027-x

**Published:** 2016-04-01

**Authors:** Susan Baxter, Delia Muir, Louise Brereton, Christine Allmark, Rosemary Barber, Lydia Harris, Brian Hodges, Samaira Khan, Wendy Baird

**Affiliations:** 1 NIHR Research Design Service Yorkshire and Humber, University of Sheffield, Regent Court, Regent Street, Sheffield, S14DA UK; 2 NIHR RDS YH, University of Leeds, Leeds, UK; 3 School of Health and Related Research, University of Sheffield, Regent Court, Regent Street, Sheffield, S14DA UK; 4 Public involvement member, Sheffield, UK; 5 Sheffield Teaching Hospitals NHS Foundation Trust, Sheffield, UK

**Keywords:** Patient and public involvement, Public involvement, Lay representation, Consumer involvement, Qualitative, Document analysis, Research design, Patient and public engagement, Participatory research

## Abstract

**Plain English summary:**

The National Institute for Health Research (NIHR) Research Design Service (RDS) for Yorkshire and Humber has been running a public involvement funding scheme since 2008. This scheme awards researchers a small amount of money to help them get involvement from patients and/or the public. Involvement activities take place at the time when researchers are planning studies, and when they are completing application forms to request funding for a proposed research project. After the public involvement activities researchers are asked to write a report for the RDS describing what they did with the public involvement funding.

This study analysed those reports using an approach which included members of a public involvement panel in the data analysis process. The aim of the work was to see what the views and experiences of researchers who received funding were, and what might be learned for the future of the scheme. Twenty five reports were analysed. Four main themes were identified, these described: the added value of public involvement; aspects to consider when planning and designing public involvement; different roles of public contributors; and aspects of valuing public member contributions. The group approach to analysis was successful in enabling involvement of a variety of individuals in the process. The findings of the study provide evidence of the value of public involvement during the development of applications for research funding. The results also indicate that researchers recognise the variety in potential roles for the public in research, and acknowledge how involvement adds value to studies.

**Abstract:**

**Background**

A regional Research Design Service, funded by the National Institute for Health Research, introduced a small grant in 2008, to support public involvement (often known as patient and public involvement [PPI]) activities during the development of applications for research funding. Successful applicants are requested to submit a report detailing how the grant money was used, including a description of the aims and outcomes of the public involvement activities. The purpose of this study was to analyse the content of these reports. We aimed to find out what researcher views and experiences of public involvement activities were, and what lessons might be learned.

**Methods**

We used an innovative method of data analysis, drawing on group participatory approaches, qualitative content analysis, and Framework Analysis to sort and label the content of the reports. We developed a framework of categories and sub-categories (or themes and sub-themes) from this process.

**Results**

Twenty five documents were analysed. Four main themes were identified in the data: the added value of public involvement; planning and designing involvement; the role of public members; and valuing public member contributions. Within these themes, sub-themes related to the timing of involvement (prior to the research study/intended during the research study), and also specific benefits of public involvement such as: validating ideas; ensuring appropriate outcomes; ensuring the acceptability of data collection methods/tools and advice regarding research processes. Other sub-themes related to: finding and approaching public members; timing of events; training/support; the format of sessions; setting up public involvement panels: use of public contributors in analysis and interpretation of data; and using public members to assist with dissemination and translation into practice.

**Conclusions**

The analysis of reports submitted by researchers following involvement events provides evidence of the value of public involvement during the development of applications for research funding, and details a method for involving members of the public in data analysis which could be of value to other researchers The findings of the analysis indicate recognition amongst researchers of the variety in potential roles for public members in research, and also an acknowledgement of how involvement adds value to studies.

## Background

Public involvement (often known as patient and public involvement [PPI]) has been increasingly advocated as an essential element of high quality and clinically relevant research [1]. By involvement in this context we are referring to patients, carers and/or members of the public actively contributing to the design, delivery, management and/or dissemination of research. However, there is limited evidence available regarding the processes and impact of public involvement in research [2]. A review in 2009 [3] reported that public involvement may increase study recruitment, improve trial design and outcome measures, and benefit the people involved. A review of published examples of involvement in research design [4] found that members of the public were reported to contribute to research design by: reviewing patient information sheets/consent procedures; suggesting outcome measures; reviewing data collection procedures; and recommending timing of recruitment and follow up.

Researchers who apply for funding to carry out studies are commonly expected to demonstrate how they have involved lay individuals in preparing their applications, to outline what changes were made to the application as a result of public involvement, and to outline in detail how they will involve members of the public in the design and conduct of the research if funded [2]. The involvement of the public at an early stage in developing proposals can present a challenge to potential applicants as, without the funding being in place, there is no identified resource to draw upon to support involvement activities. Guidelines from the advisory body INVOLVE emphasise that the time, skills and expertise of members of the public should be recognised, and that accurate budgeting for costs is essential [5]. Costs may relate for example to reviewing a research proposal, attending a meeting, preparing for a meeting, or reading relevant documents.

The National Institute for Health Research (NIHR) created 10 regional Research Design Services (RDSs) to provide design and methodological support to health and social care researchers across England, when developing grant applications to the NIHR and other national peer-reviewed funding programmes [6]. The service provides advice and expertise, and builds “bridges and connections across research groups” [7] to enable high quality research proposals. As part of their role, the RDSs provide advice to researchers on public involvement at all stages of the research study. Many of the RDSs have also developed schemes whereby they offer funding to support service user involvement at the research design stage [8, 9].

The NIHR RDS for Yorkshire and Humber has an established Public Involvement funding scheme to assist researchers to gain involvement with the public during the development of research grant applications. Four calls are held each year, and the amount requested can be up to £500. Following receipt of an award, researchers are asked to write a short report outlining how the payment was used, and the extent and ways in which the public contributed to the development of the application. Researchers are provided with guidelines for the structure of the report (see Appendix). Papers describing schemes in other RDS regions [2, 8] have provided evidence of a link between public involvement and successful funding outcomes.

A previous paper which examined the Yorkshire and Humber scheme [9] provided details of the usage of the scheme, described the characteristics of applications and outcomes, and outlined three case examples of how the award contributed to successful grant applications. The purpose of the current study was to examine in detail the reports that are completed by researchers following public involvement activities. In this study we aimed to analyse reported views and experiences regarding the processes of public involvement, in order to investigate elements that might be barriers or facilitators to public involvement in research proposals.

## Methods

We intended that the analysis process should fully include public involvement, and therefore adopted a group participatory approach to analysis of the reports on public involvement events, as outlined below.

### Approach to analysis

The approach to analysis drew on techniques of qualitative content analysis and Framework Analysis. These techniques aim to derive meaning from text, by systematic classification and coding, to identify recurring patterns or themes [10]. Content analysis can be used to describe a range of analysis techniques, including a simple counting of sections of text that contains similar words or phrases [10]. Qualitative content analysis aims to go beyond counting words, to examine the language for meaning. In addition to qualitative content analysis, we drew on techniques of Framework Analysis [11] to ensure that the process of analysis was systematic and comprehensive. In this approach the data are organised into a chart of columns and rows (with each row being an individual document, and each column a theme) to assist with the identification and labelling of text into meaningful chunks.

### Method of analysis

A workshop was arranged for the purpose of analysing the reports. Four members of the public with experience of being involved in research, and five local public involvement advisers and facilitators were invited to attend. Prior to the workshop the first author (SB) sourced and read all the reports that were available. As the length of the reports ran to many pages and included information relating to background literature and financial information, an initial extraction was carried out to copy sections of the reports that were relevant to a new document to reduce the burden on public members. There remained a large quantity of text following this process, and in order to share out the workload, six individual documents with different sets of data were created. SB broadly grouped the content of the reports into: input to the research topic; input to the proposed research methods; potential roles of public contributors in the planned study; benefits of public involvement; methods to recruit public contributors; and barriers and facilitators to public involvement. Each report was sent to a pair of workshop participants (one member of the public and one public involvement staff lead) two weeks before the event, with a request to read the text and note down anything that was interesting or important.

Ten participants attended the workshop. The session began with a brief introduction and warm up categorisation activity (on the topic of food and drink) to give participants practice and confidence in grouping written text together to develop themes and sub-themes. Following this, the group divided into the pairs who had received the same text. They discussed the data and wrote down themes that they had identified on sticky notes. Key themes across all the reports were discussed as a whole group and recorded on flip chart sheets. In an interactive process of framework development participants discussed and stuck their notes on the flip chart sheets, with sub-categories/themes developed by grouping sticky notes together.

Two weeks following the workshop the completed framework of themes and sub-themes was circulated via email to attendees asking for feedback on the categories, any themes that were unclear, or did not appear to be representative of the data that they had scrutinised. SB then re-read the data from the reports to populate the framework with text, and this was re-circulated for input from the group to develop the final analysis.

## Results

We analysed data from 25 reports written by researchers following public involvement activities. The length of the documents varied from four pages to more than 20 pages, with varying level of detail. The outline framework of themes and sub-themes that was developed from analysing these reports is presented in Table 1. As can be seen, there were four key themes identified relating to: the added value of public involvement; planning/designing involvement; intended roles of public members; and valuing the contributions of public members. The sub-themes were commonly grouped by the phase in the research process: either prior to the study commencing; during the study; or after the study. Each of the themes will be briefly described with illustrative quotations from the reports provided.

**Table 1:** Framework of themes developed from the data

Added value of public involvement	*Prior to study commencing*	Validating ideas
		Input in regard to appropriate terminology
		Developing interventions
		Ensuring clarity/user friendliness of written information
		Ensuring appropriate outcomes
		Acceptability of data collection methods/tools
		Alerting to potential ethical/patient safety issues
		Advice regarding research processes such as recruitment/drop out/follow up
	*During study*	Ensuring research conducted with sensitivity/empathy
		Sharing experiences/emphasising value of individual experiences in study
Planning/designing public involvement	*Prior to study commencing*	Recruitment to public involvement event
		Timing
		Venue
		Training/support
		Payment
		Format of sessions
		Facilitation skills
	*During study*	Training/support
		Recruitment to study advisory groups
		Payment
		Contacting members
		Commitment/burden
	*After study*	Feedback to members on outcome/findings
Role of public members	*Prior to study commencing*	Proposal development
		Co-applicant
		Feedback on accessibility of language
	*Intended during study*	Advice
		Public panel
		Project steering group/management panel
		Scrutinise conduct of research
		As patient interviewers
		Involvement in analysis and interpretation
		Review of documentation to be used
		Assist with ethical approval documentation
		Consult regarding any issues regarding recruitment/involvement
		Help with any developing or evolving issues
	*Intended after study*	Assist with dissemination
		Assisting with writing up findings
		Assist with translation into practice
Valuing public member contributions	Recognising expertise of members	
	Feeding back	
	Value for public members	

### Added value of public involvement

In the reports researchers described how involvement activities had been valuable prior to the study commencing. The added value had been in terms of validating or adding to their knowledge and perceptions of the intended research subject area:

“They felt that research attempting to understand what people can do to maintain wellbeing would be very beneficial to patients”.

“Comments were made that it [the research topic] resonated very strongly with their own experiences”.

There were examples of how public involvement had directly influenced the particular area of the research proposal:

“It prompted us to think we should focus, in our proposal, only on improving post-diagnostic support, since this was a more discrete issue”.

Public involvement was also reported to have been valuable in developing the specifics of an intervention that was going to be tested. Researchers had received valuable feedback in regard to: who should deliver a programme; the content of an intervention; and the acceptability of a programme to patients:

“Based on these suggestions, I decided the next step will be to modify these relaxation methods” (the content of the relaxation tape provided to participants was changed).

“The intervention would be acceptable to patients”.

Public contributors had also provided valuable advice in regard to ensuring that outcomes of interventions were of importance to patients:

As a consequence, we have included this as a secondary outcome in the plan of care that all patients recruited into the study would receive” (an additional wellbeing outcome was included).

A key area for input was ensuring the appropriateness of terminology, clarity and user friendliness of written information, such as the Lay Summary and the patient information leaflet:

“Participants recommended some word changes and alternative phrasing to ensure it is fully understood by patients”.

“Other suggestions have been incorporated into a revised version of the Lay Summary”.

The methods and tools for collecting outcomes was another area of important contribution. The researchers used the opportunity to try out completion of questionnaires, or asked for views on the acceptability of measures and methods to collect the data:

“From talking to attendees I understood how people had very different views on using outcome measures…therefore in the research proposal I have developed flexibility in the process of testing using outcome measures”.

“All patients were willing to undergo the extra tests”.

During these discussions some researchers reported that potential ethical or patient safety issues were raised, such as how abnormal test results should be communicated.

Advice had been received regarding research processes such as methods for recruitment, measures that could be taken to minimise drop out, and the length of time that patients should be followed up:

“As a direct result of discussions the focus of recruitment was altered, this had a sizeable impact on the design of the trial and altered the aims and objectives of the project”.

“There was a strong preference for recruitment to be initiated via a face-to-face approach in clinic because this would allow patients to ask questions about the study straight away”.

“All parents agreed that ideally the study follow up period should be at least 12 months because of changes over a 6–12 month period in a child’s life that can influence the sleep pattern”.

“All agreed they would rather complete the questionnaires at home”.

Researchers reported that public involvement had highlighted the need to ensure that studies were conducted with sensitivity and empathy. Involvement activities had also emphasised the importance of capturing varying individual experiences of healthcare in order to inform the study design.

As these reports were written prior to research studies commencing, this explains the lack of data reporting researcher perceptions of added value after a study. However, intended roles for public members within studies were described, and these data are outlined within the theme of public member roles below.

### Planning/designing public involvement

The reports provided insight into strategies that are important to consider when planning public involvement. Researchers described methods that they had used to recruit potential attendees to their involvement events/activities. These included using key representatives or healthcare professionals from local services to approach individuals known to them, attending formal and informal meetings, using online forums, and placing adverts on websites or in NHS departments. Several of the researchers described challenges to recruitment, with also significant drop out of attendees immediately before the event:

“We had originally planned to involve 6–8 patients in this PPI work. Due to recruitment difficulties we only ended involving four patients. In the future, we will allow more time for recruitment and identify patients from more than one surgeon’s clinic”.

“Some families could not take part in our discussion groups as they were unable to make care arrangements for their other children. In addition, we also received late notice cancellations due to illness and other engagements”.

There were recommendations that potential issues of timing and location such as school holidays, travelling distance, and start time should be considered carefully. Also, that a longer meeting, with time for small group work might be useful:

“Arranging the session in the holiday period probably accounted at least partly for the low attendance”.

“Maybe being longer as I felt there was a great deal more we could have discussed”.

Elements which contributed to successful meetings were: sending hand-outs to participants beforehand; having an agenda; ensuring that translators were available if required; having a relaxed atmosphere where all could contribute; and considering whether several smaller groups might be preferable to one larger event:

“People would have liked to have seen the hand-outs before the meeting”.

“A member of staff or the researcher translated the meeting content for those participants and service users that did not speak English”.

“They were encouraged to contribute to discussion, and during the meeting their views were valued”.

“Two separate PPI meetings were convened to minimise the anxiety and stress”.

The importance of having well-developed facilitation skills was mentioned, in order to ensure that all participants had a chance to contribute, and to create an atmosphere that was enjoyable and interesting.

An event prior to submission of the research proposal was described as a useful avenue for finding members for advisory groups during the study. The need to consider training and support for public contributors during the study was also highlighted, together with the importance of planning how any payment for time and expenses would be provided:

“A range of training would be needed including interpersonal skills and self-confidence in addition to training on research processes and skills”.

“Payment for time and out of pocket expenses will be provided”.

“Participants expressed suspicion of the idea of payment for participation” (involvement in advisory groups).

Researchers found that email was a popular way of communicating with public contributors, with some also recommending the use of texting to send reminders about meetings.

### Role of public members

A range of planned or intended roles for public contributors was described before, during and after a study. As outlined above in the section on the added value of public involvement, feedback gained at involvement events made significant contributions during development of the research proposal.

“I used this feedback to improve the application”.

“The research team are confident that the bid has been improved by the rich data collected during this meeting”.

Some researchers used a second round of consultation after the event to gain feedback on the changes that they had made:

“A second meeting was held, after a draft grant application and copies of the data collection tools had been circulated, to obtain feedback on specific aspects of the study design and grant application such as the proposed recruitment strategy, the choice of outcomes, and the content of the plain English summary”.

Another potential role for public members prior to a study was as a co-applicant. The possibility of this was mentioned by three researchers, although none of the reports described public members taking on this role.

Intended roles during the study described were: acting as general advisers; becoming a member of a public advisory group/panel; or being a contributor on the project steering group:

“We plan for two patients from the PPI group to attend each trial management group meeting”.

“We also explained our plan for patient involvement activities if the project is funded, and invited the participants to form a patient involvement group that would support the research study”.

Part of the role of these individuals would be to oversee the conduct of the research:

“To scrutinise the conduct of the research, ensure that resources are used appropriately and to monitor progress”.

Patient and public involvement was also planned during the study to contribute to analysis and interpretation of the data; to review documentation used during the study and assist with ethical approval documentation; to seek advice regarding recruitment/involvement; and to help with any developing or evolving issues:

“We intend to involve one or two of the participants from this focus group in the design of the trial; especially in the formulation of information sheets and consent forms”.

“Having pregnant women on the panel will enable us to discuss and address issues around recruitment and concordance from their perspective. This will enable us to alter or improve recruitment strategies”.

After the study it was envisaged that public members would assist with dissemination, with writing up findings, and with translation of the findings into practice:

“The planned events are designed to engage a wide range of stakeholders and will form a key part of our definitive trial proposal development, dissemination, and translation into practice plan”.

The importance of discussing and managing the commitment/burden for members of the public during a study was emphasised. It was suggested that fluid membership might be considered, or several individuals could share the role between them in order to reduce time burden. It was acknowledged that ill health could limit the ability of some individuals to make a time commitment:

“The original PPI group felt it would be acceptable to have a fluid membership of pregnant women in the PPI group”.

“They agreed that having more than one expert patient on the project group meant individuals could ‘share the load’ and liked the potential for ‘peer support’ between expert patients”.

### Valuing public member contributions

The perception of public contributors as being individuals with expertise to share, was a thread throughout the data:

“Interestingly our fears [regarding the potential for distress to participants] were largely unsupported by the expert patients. Instead the expert patients raised other issues they felt to be of greater importance”.

This recognition of expertise and value was illustrated clearly in researcher comments regarding changes that were made following public input. The importance of providing feedback to participants following events was also highlighted:

“I have assured the panel that all comments have been reflected upon and the comments have been included in the submission”.

“A lay summary document was produced which detailed the key messages to be taken from the discussion and how the proposed research will be developed as a result”.

Some of the reports included feedback from participants who had taken part in the involvement events. These data included perceptions that being involved was of value to members of the public themselves, and provided insight into the motivation for them taking part:

“I felt good in the meeting and it gave me a chance to get to know people with these issues; and it gave me an insight into how other people are and I got to talk openly, as well as listening to other people’s feedback”.

“This is certainly helping me anyway, being part of this…knowing that it’s not just me in this world that it affects”.

“I hope my input was helpful for all the (those) involved”.

## Discussion

This analysis of reports written by researchers following public involvement activities in developing applications for research provides evidence regarding the perceived value of involvement during the research design process. The views and experiences of researchers described in this study provide further data to support the value of involvement of patients and the public at an early stage in developing proposals.

The research findings are also consistent with existing guidance for researchers on public involvement issued by organisations such as INVOLVE [12], and the Research Design Services [13]. The results of the study provide a useful framework for researchers to consider public involvement at all stages when developing proposals, and enable them to draw on the experiences of other researchers. The group method that we used to carry out the data analysis was a useful approach to achieving involvement of individuals who have no previous experience of qualitative data analysis. The potential for lay individuals to contribute to data analysis is commonly recommended [12–14]. However, we have found few detailed examples of studies reporting methods used to facilitate members of the public contributing to data analysis (a notable exception is described by Gillard et al. 2012 [15]).

The Framework Method has been suggested as being appropriate for use in research teams where not all members have previous experience of conducting qualitative research [16], and our experience concurs with this. Our data related to a discrete topic, which may have made the process of analysis more straightforward. Other researchers have cautioned that the Framework Method deals less well with highly heterogeneous data [16].

A short debrief was held at the end of the analysis workshop, where participants were encouraged to reflect on the participatory process used. All participants valued the warm up exercise where they were asked to categorise different types of food as this demonstrated the categorisation process in an accessible way and helped to build confidence.

Group members reported that working in pairs to look at specific sections of data worked well. Pairs reported that each person brought slightly different insights and perspectives to the data. Breaking the data into manageable sections was also appreciated, as this made both the workshop tasks and the preparatory work more manageable. Having one person who had read all the reports in full provided valuable oversight, and meant that individual sections could be put into context if needed. One potential negative aspect of splitting the text into sections before the workshop, is that the data had already been filtered by a researcher, which could impact on people’s interpretations. Feedback from participants following the workshop was that reading larger sections of data would have been overly onerous, and that different interpretations of data were still possible from reading and discussing each other’s sections.

Everyone present had some previous experience of public involvement. This was positive in that it allowed the group to easily understand the topic and the aims of the project. However, this also created a challenge in that people moved easily into discussing their own experiences, and at times made some assumptions about the activities being described in the reports. Workshop participants had to challenge each other regarding this, and bring discussions back to the data. The group had worked together in the past, and therefore felt able to challenge each other when needed. Group members reported that this would be more difficult with a group who did not have a prior relationship. A learning point here, is that a certain amount of preparation and relationship building is necessary within a workshop where people are coming from different backgrounds, and where challenging each other’s perceptions is central to the process.

### Limitations

We recognise a limitation of the study is that the data came from researchers who had applied for, and achieved funding for public involvement activities. Their views and perceptions may therefore be more positive than researchers who have not recognised the potential benefits, or have not considered public involvement. The researchers were also responding to their funder, and may therefore be more inclined to be positive. The data were also analysed by a group of people who already had public involvement experience, and pre-existing views and experiences may have influenced their interpretation of the data.

It is also important to note that while the reports provide evidence regarding how involvement activities had contributed to development of the research proposal, the potential expected impact on the study itself (if it was successful in receiving funding) was based on researcher intentions rather than actual experience.

### Future work

This work provides evidence of the value of public involvement in the development of proposals however, the impact during studies themselves requires further investigation. It would be helpful for example to gain a further understanding regarding how PPI elements of proposals have informed funding decisions, and how the intentions for PPI of researchers prior to a study, related to the actual involvement achieved. Research examining the implementation of plans for PPI in clinical trials [17] reported that while most of the plans for PPI had been implemented, some researchers spoke of using it as a means of “ticking the right boxes” during the application process. This study also found considerable variability regarding the extensiveness of planned PPI activity, and varying clarity with which plans were described. This lack of consistency regarding the quality and implementation of PPI in studies which have been accepted for funding, suggests that further investigation of PPI elements throughout the entire course of a research project would be valuable.

A recently completed study [18] has provided evidence that PPI during studies can have positive outcomes in the form of changes to study design, and improvements to recruitment and dissemination when enabling contexts are present. As the authors of this study suggest, it would be interesting to track PPI involvement not only through the course of a project, but also from the earliest stage of research proposals to longer term impact on practice.

Following examination of reports the RDS has continued its consideration regarding ongoing delivery of the funding scheme, and its evaluation [9]. There are plans to follow up a sample of the award recipients including members of the public who had been actively involved in the research, to explore what happened later in the process, and whether the planned public involvement happened when studies commenced. We are also considering ways to seek views on the involvement activities from funders.

The findings of our study indicate that, while public involvement is highly valued, there may be a need for greater support regarding carrying out involvement activities, and the best methods for accessing potential lay members. Some researchers described the need for skills in facilitation, as the public involvement process was very different to their routine clinical interactions, for example one researcher noted “I need to improve my facilitation skills to learn how to better manage more dominant members of the group.” The RDS is considering how best to provide additional advice and support in order that researchers have skills to make the most of public involvement opportunities. For example information on how to structure sessions, examples of questions that could be asked, tips on managing group discussions, may assist researchers in preparing for sessions.

## Conclusions

The findings of the study provide evidence of the value of public involvement during the development of applications for research funding. The results also indicate recognition amongst researchers of the variety in potential roles for the public in research, and the acknowledgement of how involvement adds value to studies. The use of the participatory approach to analyse the reports that we describe could be of value to others wanting to carry out collaborative data analysis.

### Ethics approval

Ethical approval was not required as the study analysed anonymous documentary data only.

## Appendix. Guidance on structure of the report

### Introduction

As stated in your funding approval letter, the NIHR Research Design Service for Yorkshire and the Humber would like to receive a short report detailing how the award was used, the extent and ways in which the public contributed to the development of the grant application, and whether the grant application was successful. We would also like this report to include a short contribution from a member of the public that you involved in the design process, reflecting on their involvement in, and contribution to, the process. This document is to provide you with guidance on what to include in your report.

### Structure

• Introduction−background to the grant application or reasons why the panel was created

• Aim−why you applied for the award, what you wanted patients and the public to do

• Method: how you went about identifying and recruiting relevant patients and the public to take part, and a description of how you engaged with them (e.g. did they take part in consultation meetings/focus groups, or bid-writing meetings etc.)

• Description of the type of people who took part, and how many people took part in each relevant involvement activity

• Description of the contributions made by the patients and the public, and a consideration of what was changed or adapted as a result of their involvement

• A discussion of how you evaluated the involvement of the patients and the public, together with a short account from at least one person you involved wherever possible

• (for project-specific applications) A consideration about how patient and public involvement will be taken forward should you be awarded funding

• A consideration of any difficulties encountered and if there are things you might have done differently

• (for project-specific applications) Notification of whether or not you were successfully awarded funding or the date of when you expect to hear confirmation

• Appendix: detailed breakdown of how the funding was spent
